# Cultural-morphology and molecular analysis of *Botryodiplodia theobromae*, a pathogen of coconut fruit

**DOI:** 10.12688/openreseurope.19692.2

**Published:** 2025-11-12

**Authors:** Osayomore Endurance Ekhorutomwen, Safa Oufensou, Nnamdi Ifechukwude Chidi, Quirico Migheli, Olalekan Hakeem Shittu

**Affiliations:** 1 Plant Pathology Division, Nigerian Institute for Oil Palm Research, Benin City, Edo, 302115, Nigeria; 2 Department of Agricultural Sciences and Desertification Research Centre, University of Sassari, Sassari, Sardegna, 07100, Italy; 3 Department of Plant Biology and Biotechnology, University of Benin, Faculty of Life Sciences, Benin City, Edo, 300213, Nigeria

**Keywords:** Botryodiplodia theobromae, coconut, fruit rot, premature nut fall, culture, primers, bioinformatics.

## Abstract

**Background:**

In coconut production, less than one third of the button nuts produced in an inflorescence eventually develop into mature nuts, as a result of fruit rot and premature nut fall diseases. *Botryodiplodia theobromae* is linked to the fruit rot and premature nut fall diseases of coconut, resulting in consequential yield loss of over 60%. Furthermore, the fungus poses a concern to plants, farmers, and plant health authorities because it may survive in plant materials as an endophyte, escaping the quarantine process. This study was carried out to analyse the cultural, morphological, and molecular characters of *B. theobromae* associated with these diseases in coconut fruits.

**Methods:**

Eight isolates of *B. theobromae* used in this study were collected from diseased coconut fruit samples (showing signs of rot and freshly fallen nuts) in two major coconut producing areas in Nigeria. Pure culture of isolates were obtained using potato dextrose agar (PDA) medium. The culture medium and microscopy were used to also examine the cultural and morphological characters of isolates. For molecular characters of isolates, DNA was extracted from each isolate and amplified with a polymerase chain reaction (PCR) using a universal primer (ITS1F/4R) and three specific primers (Lt347-F/R, Bt2aF/bR, and EF1-728F/EF2-728R). Furthermore, PCR amplicons obtained from the ITS1F/4R primers were sequenced and analyzed using bioinformatics and phylogenetic methods. The genetic similarity and variation of isolates were also determined.

**Results:**

The colony texture/color, ITS1/4 gene sequence information and phylogenetic analysis clustered the eight isolates of *B. theobromae* into five categories. The ITS1/4 sequence information using a blast search in the NCBI database, confirmed all eight fungal isolates as *B. theobromae*.

**Conclusion:**

This study has provided a guide for proper categorization of *B. theobromae*, that is a prerequisite for early diagnosis and management of this pathogen in coconut producing areas.

## Plain Language Summary

This study provides information on the identification of the pathogen causing fruit rot and premature nut fall diseases of coconut.

## Introduction

Coconut ( *Cocos nucifera* L.) is a perennial plant grown in over 90 countries around the world, by an estimated 11 million farmers across 12 million hectares of land ( FAO, 2014; Gurr *et al*., 2016). Coconut serves as food and a cash crop in many developing countries, providing a several foreign exchange to nations cultivating the palm ( Bourke & Harwood, 2009). Coconut production in Nigeria are mostly affected by soil, climate and diseases ( Ekhorutomwen *et al*., 2019). In coconut production, less than one third of the button nuts produced in an inflorescence eventually develop into mature nuts, that is, as a result of fruit rot and premature nut fall diseases ( Ekhorutomwen *et al*., 2024; Rajapakse *et al*., 1995). In addition *, Botryodiplodia theobromae* (a cosmopolitan fungal pathogen) is associated with the fruit rot and premature nut fall diseases of coconut, resulting in consequential yield loss of over 60% ( Dheepa *et al*., 2018; Marques *et al*., 2013; Taylor & Hyde, 2003). *B. theobromae* can occur in nature as a parasite, saprophyte, or endophyte ( Alves *et al*., 2008; Machado *et al*., 2014; Slippers & Wingfield, 2007). The fungus poses a concern to plants because it may survive in plant materials as an endophyte, escaping the quarantine process ( Dheepa *et al*., 2018). The fungus has a wide host range, including gymnosperms and angiosperms ( Alves *et al*., 2008; Machado *et al*., 2014). Factors that contribute to the distribution of *B. theobromae* causing fruit rot and premature nut fall diseases of coconut include; host plant range determined by transmission from infected palm to susceptible healthy palm, *B. theobromae* preference for host palm, variation in transmission or infection rate, virulence of *B. theobromae*, poor agronomic practice, and climate (weather) factors such as temperature, rainfall, wind, relative humidity, etc ( Ekhorutomwen *et al*., 2019).

The appearance of symptoms of fruit rot and premature nut fall diseases in coconut palms, together with the emergence of numerous *Botryodiplodia* species and other fungi species associated with coconut has called for precise and accurate identification of *B. theobromae* as the causal agent of fruit rot and premature nut fall diseases in coconut ( Dheepa *et al*., 2018; Munirah *et al*., 2017). As the species covered in this article, *B. theobromae* is the recognized scientific name; it supersedes both the teleomorph *Botryosphaeria rhodina* (Berk and M.A. Curtis) Arx and the synonym *Lasiodiplodia theobromae* Pat. The information on population of plant pathogen(s) will be used for developing strategies to increase the durability of crop varietal resistance towards such pathogen(s) ( Xia *et al*., 2000). Besides improving the knowledge of pathogen(s) causing diseases in coconut, accurate identification of pathogen(s) is important to establish proper control measures, quarantine programs, and to develop resistant crop varieties to pathogen(s) ( Asman *et al*., 2024; Munirah *et al*., 2017; Ramachandran *et al*., 2015). In the past few years, the advancement of molecular techniques has yielded various insights about the population of pathogens ( Séré *et al*., 2007). Several molecular methods have been used to identify and reveal genetic similarity or polymorphism within populations of plant pathogenic fungi ( Hernández-Ceja *et al*., 2021; Kang *et al*., 2002). The genetic makeup of *B. theobromae* has been examined using a variety of molecular tools ( Gunamalai et al., 2023; Santos et al., 2017). These include, but are not limited to, the use of the following genetic identifiers: the translation elongation factor gene, the tubulin gene, and the internal transcribed spacer domain.

The internal transcribed spacer and tubulin gene have been utilized to identify *B. theobromae* isolated from mango, cashew, and other tree crops ( Munirah *et al*., 2017; Pandey *et al*., 2024). The pair of primers often used to amplify the internal transcribed spacer and tubulin gene in a PCR are ITS1/4 with F: 5’-TCCGTA GGTGAACCTGCGG-3’ and R: 5’-TCCTCCGCTTATTGATATGC-3’ for ITS, while Bt2a/b with F: 5′-GGTAACCAAATCGGTGCTGCTTTC-3′ and R: 5′-ACCCTCAGTGTAGTGACCCTTGGC-3′ for tubulin gene ( Xie *et al*., 2016). Inter-simple sequence repeat, simple sequence repeat, and random amplified polymorphic DNA identifiers have also been used to describe *B. theobromae* obtained from Morus alba and Agave sisalana ( Cardoso & Wilkinson, 2008; Xie *et al*., 2016). Ten tiny gene satellites, or simple sequence repeats, were developed by Cardoso and Wilkinson (2008) in order to genetically characterize *B. theobromae*. Shah *et al*. (2010) also developed nine tiny gene satellites and seven random amplified polymorphic DNA identifiers, which they used to characterize *B. theobromae* isolated from the pear trees. Santos *et al*. (2017) and Jacobs *et al*. (2004) developed translation elongation factor with pair of primers: EF1-728F (5'-CATCGAGAAGTTCGAGAA-3') and EF2-728R (5'-GGARGTACCAGTSATCATGTT-3') for the molecular identification, sequence, and phylogenic analyses of *B. theobromae* isolated from cashew, native bark beetles, and Tomicus piniperda. Xu *et al*. (2016) developed PCR-based processes that utilize three specific primers, namely, primers Lt347 with F: 5'-AACGTACCTCTGTTGCTTTGGC-3' and R: 5'-TACTACGCTTGAGGGCTGAACA-3', Np304 with F: 5'-TGAACTTCGCAGTCTGAA-3' and R: 5'-CTCCAAAGCGAGGTGTTT-3', and FaF/Bt2b with F: 5'-CATCCGCAGCGTGGGAGAACAT-3' and R: 5'ACCCTCAGTGTAGTGACCCTTGGC-3') for identification of the species belonging to the Botryosphaeriaceae family linked with stem blight disease of blueberry in China ( Xu *et al*., 2016).

In this study, the universal primer (ITS1F/4R) and three species specific primers (Lt347-F/Lt347-R, Bt2aF/Bt2bR, and EF1-728F/EF2-728R) were used to amplify the DNA of *B. theobromae* isolated from diseased coconut. Furthermore, the amplification of the ITS1/4 region, together with the sequencing of the ITS1/4 region in conjunction (with other molecular identifiers), cultural and morphological characters, was employed in this investigation to identify *B. theobromae* isolated from diseased coconut fruits with signs of fruit rot and premature nut fall ( Ekhorutomwen *et al*., 2024; Pandey *et al*., 2024). Hence, with economic importance of fruit rot and premature nut fall diseases of coconut, this study was carried out to analyse the cultural, morphological and molecular characters of *B. theobromae* causing these diseases in coconut fruits.

## Methods

### Culture media

Potato dextrose agar (PDA), Czapek Dox agar (CDA), and sodium hypochlorite were purchase from Sigma-Aldrich, St. Louis, MO, USA, and used for the study.

### Study location

Two (2) locations namely; NIFOR Main Station and Coconut Garden, Isihor, in Ovia North East Local Government Area, Edo State, Nigeria, were used for this study.

### Source of coconut fruits

Diseased coconut fruits showing signs of rot and freshly fallen nuts were collected from coconut plantations cultivated with the following coconut varieties {that is, green dwarf (GD), orange dwarf (OD), yellow dwarf (YD), red dwarf (RD)} in the chosen location.

### Isolation of fungal from diseased coconut fruits

Diseased fruit samples (showing signs of rot and freshly fallen nuts) were taken to the laboratory with a transparent screw cap container ( Phipps & Porter, 1998). Sterile knife was used to dissect diseased coconut fruit samples, and the dissected samples were surface sterilized with 200 ml 0.5% sodium hypochlorite (hypo; 3A25N). Thereafter, 1000 ml each of potato dextrose agar (PDA; ISO 9001) and Czapek’s Dox agar (CDA; ISO-9000-69-5) were prepared separately, sterilized with an autoclave, and later dispensed into petri dishes. A sterile inoculating needle was used to inoculate the dissected samples into petri dishes containing PDA and CDA, and incubated at room temperature for 7 days ( Dheepa *et al*., 2018; Phipps & Porter, 1998). Stock cultures of fungal isolates were obtained using the hyphal tip transfer procedure and maintained in tube slants of PDA at 10ºC ( Phipps & Porter, 1998). The growth of fungal isolates on petri dishes containing PDA and CDA were used for colony description ( Ashokkumar *et al*., 2018; Osagie *et al*., 2013). Colony description together with microscopy observation were used for presumptive identification of each fungal isolates as described by Commonwealth Mycological Institute (CMI) manual ( Ashokkumar *et al*., 2018; Dheepa *et al*., 2018; El-Morsi & Ibrahim, 2012; Osagie *et al*., 2013; Shah *et al*., 2010). Thereafter, the fungal isolates were confirmed using molecular techniques ( Ashokkumar *et al*., 2018; El-Morsi & Ibrahim, 2012; Shah *et al*., 2010).

### Colonial morphological (macro and micro) characterization of fungal isolates

With the aid of a sterile cock borer, nine-millimeter discs from margins of actively growing 7-day-old culture of eight fungal isolates were plated on the center of 9 cm Petri dishes containing PDA medium. Each isolate was replicated three times. Colony growth rate was recorded by measuring colony diameter after 24 and 48 h of incubation, while colony color and texture observations were recorded after 48 h of incubation ( Adeniyi *et al*., 2016; Dheepa *et al*., 2018; El-Morsi & Ibrahim, 2012; Shah *et al*., 2010). Pycnidiospores characters of each isolate *viz*., day of production, number, arrangement, size, and color were observed at weekly interval with the aid of a simple microscope.

### Identification fungal isolates with molecular tools

With the use of the Fungal DNA Isolation Kit, DNA was extracted from the mycelia of the eight fungal isolates. The isolated DNA was purified and thereafter amplified using the fungal universal primer (ITS1F/4R) and three specific primers (Lt347-F/R, Bt2aF/bR, and EF1-728F/EF2-728R) in a PCR ( Cardoso & Wilkinson, 2008; Hernández-Ceja *et al*., 2021; Wang *et al*., 2011; White *et al*., 1990; Xie *et al*., 2016; Xu *et al*., 2016). The DNA amplicons from PCR were cleansed with the help of a QIA-quick purification kit. The amplicons were separated with gel electrophoresis ( Cardoso & Wilkinson, 2008). The purity of the extracted DNA were checked with the aid of a nanodrop spectrophotometer ( Table 5). Also, the amplicons from the ITS1F/4R region were sequenced using a cycle-sequencing kit in a sequencing machine (ABI PrismTM) ( Cardoso & Wilkinson, 2008). The base sequence from the ITS1F/4R region was edited and analyzed (to determine the similarities between the strands) using multiple sequence alignment, and thereafter, it was blasted using a blasting tool in the NCBI database for identification of each isolate ( Cardoso & Wilkinson, 2008; Disegha & Akani, 2019).

### Bioinformatics and phylogenetic analysis of fungal isolates

DNA fragments that were generated from PCR using the primer (ITS1F/R) and a genotyper were used to generate data that were transformed into a binary character matrix by scoring the presence or absence of each nucleotide base at each locus (that is, “1” for the presence and “0” for the absence of a nucleotide base at a particular position). The generated combined data set (that is, nucleotide sequence information or codons) from the ITS1F/4R primer obtained from the DNA of the eight fungal isolates was aligned with the ClustalW algorithm, and dendrograms were also made using the same ClustalW algorithm in MEGA X ( Excoffier *et al*., 2007; Kumar *et al*., 2018; Shah *et al*., 2010).

### Data analysis

DNA profile that was generated using a molecular marker (ITS1F/4R) was analyzed using MEGA X ( http://www.megasoftware.net) ( Cardoso & Wilkinson, 2008; Kumar *et al.*, 2018). While data obtained from fruit rot and premature nut fall diseases were calculated using the percentage of disease incidence (PDI) in a Microsoft Excel worksheet ( Ashokkumar *et al*., 2018; Dheepa *et al*., 2018).

## Results

### Colonial morphological (macro and micro) characterization of *B. theobromae* isolates

**Figure 1.**
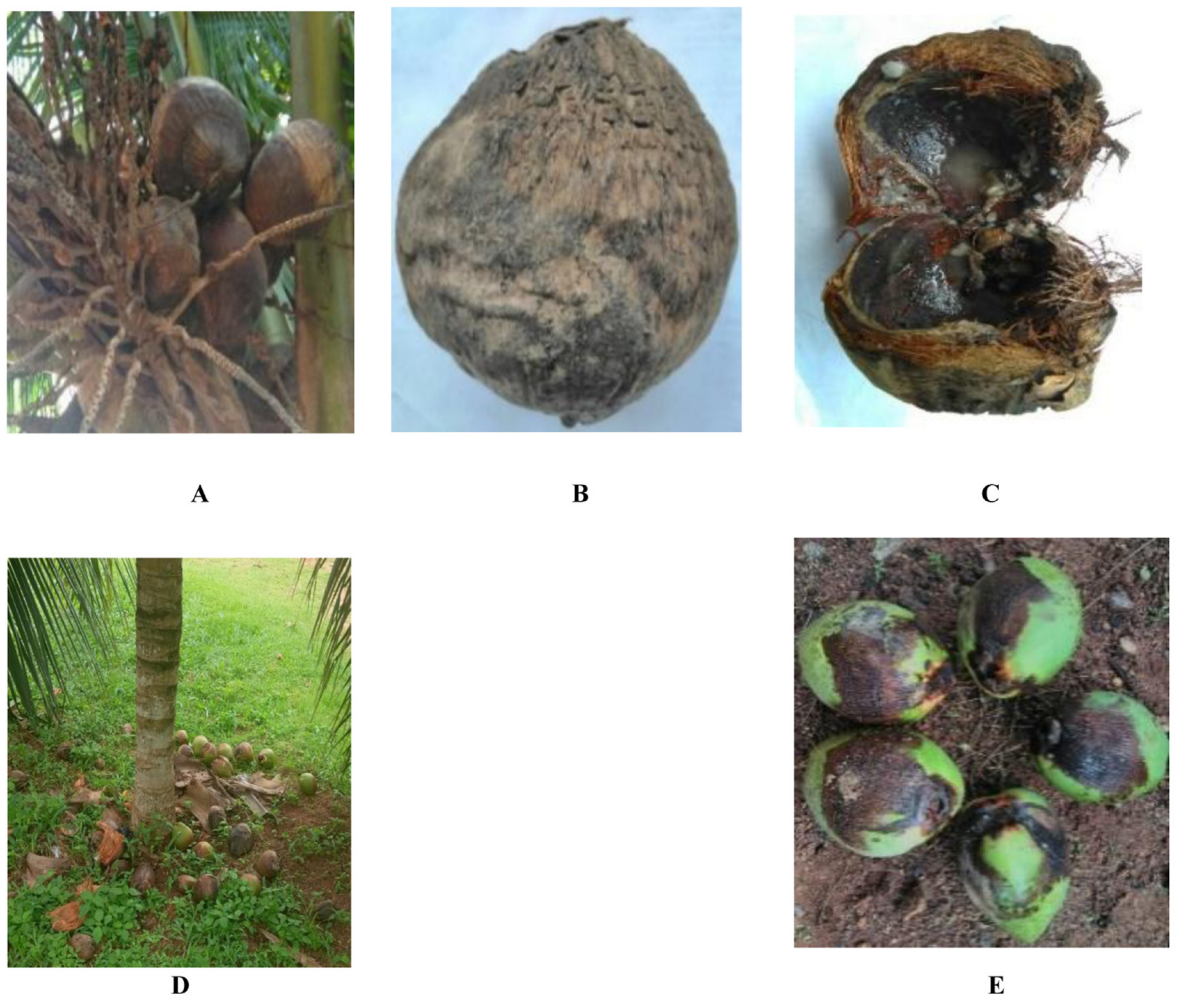
Signs and symptoms of coconut fruit rot and nut fall: **A** - **B**: Green dwarf coconut fruit with rot; **C**: dissection of green dwarf coconut fruit with rot; **D**: immature fallen nuts around the base of coconut palm; **E**: Cluster of fallen nuts of mature GD coconut with extensive rot.

**Figure 2.**
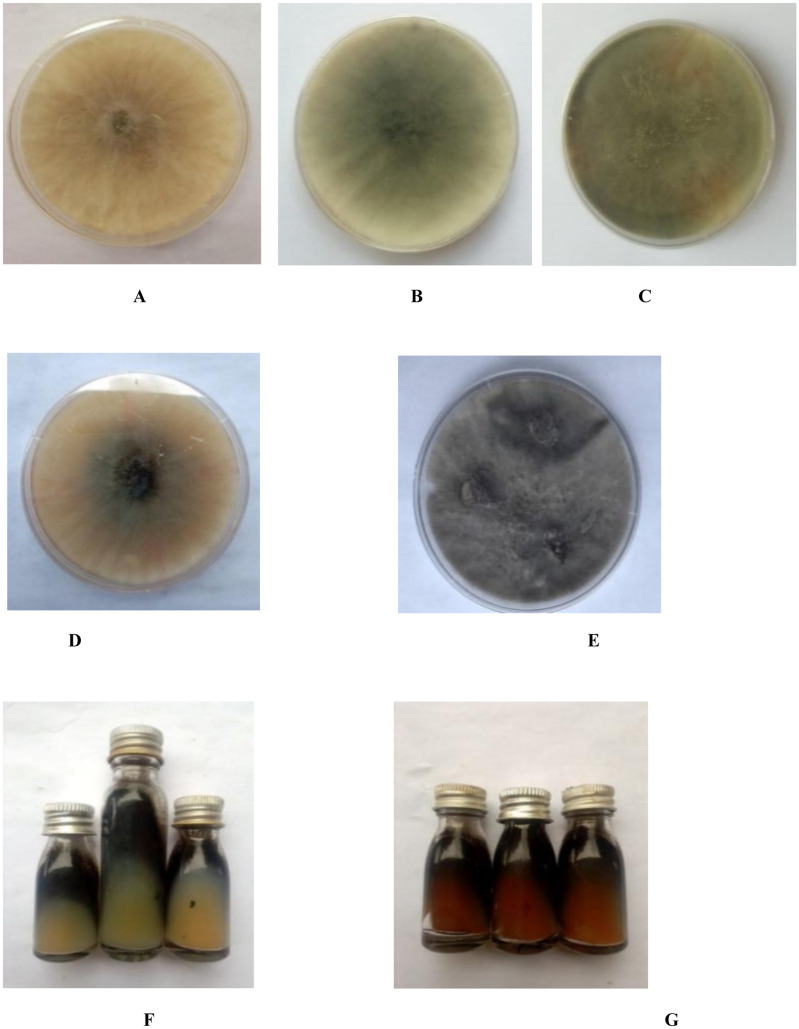
**A**- **G**: Isolates of *Botryodiplodia theobromae* colony on potato dextrose agar in Petri dishes and slant.

**Figure 3.**
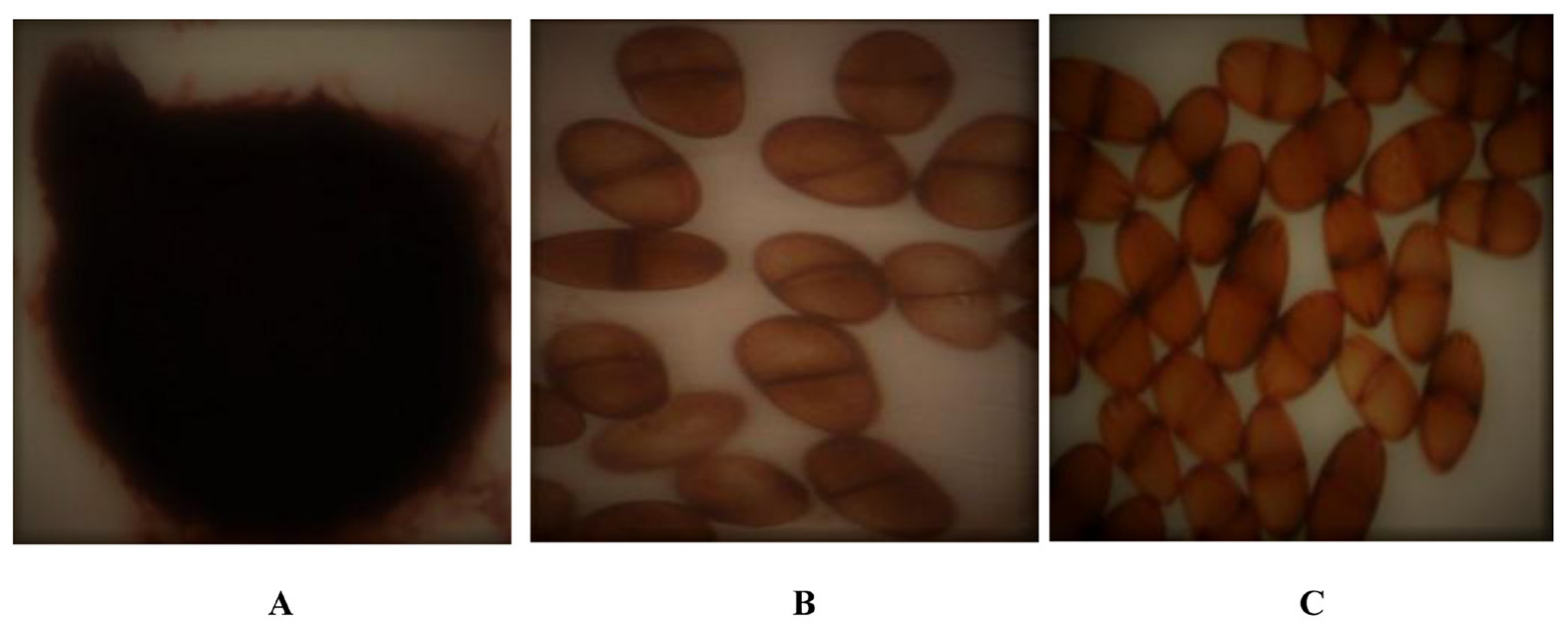
*Botryodiplodia theobromae* conidium and conidiospore; **A**: Conidium, **B**: Scattered conidiospore, **C**: Clustered conidiospore.

All eight isolates of *B. theobromae* were coded as Bt.NGD, Bt.NOD, Bt.NYD, Bt.NRD, Bt.IGD, Bt.IOD, Bt.IYD, and Bt.IRD in this study. 等线 Figure 1 and Table 4 show fungal pathogen was isolated from diseased coconut fruits showing signs of rot and fallen nuts ( Phipps & Porter, 1998). The eight isolates of *B. theobromae* isolated from diseased coconut fruits with respect to their colonial morphological characters, show varying colony texture and colour on PDA after 72 h incubation. Colony growth rate of the eight isolates of *B. theobromae* were very high after 72 h of incubation, indicating their fast-growing nature ( Table 1 and Figure 2). The eight fungal isolates filled the entire 9 cm diameter Petri dish surface after 48 h incubation. Three out of the eight isolates (Bt.NOD, Bt.NYD, Bt.IRD) produce brownish or reddish pigmentation (botryodiplodin) after 30 days in PDA slant (Plate 1), while five out of the eight isolates (Bt.NGD, Bt.NRD, Bt.IYD, Bt.IGD and Bt.IOD) did not show pigmentation in PDA slant. Time taken for conidiospore production varied between 63 to 70 days under culture condition (Bt.NOD and Bt.IYD produced conidiospore after 63 days of incubation, Bt.NYD, Bt.IGD and Bt.IOD produced conidiospore after 70 days of incubation, while Bt.NGD, Bt.NRD and Bt.IRD did not produce conidiospore on culture media after incubation for over 70 days ( Figure 3 and Table 3). Conidiospore arrangement was clustered in Bt.NYD and Bt.IGD, scattered in Bt.NOD, Bt.IOD, and Bt.IYD. The conidiospore was light brown, initially hyaline, mostly septate, with few aseptate, and, conidiospore size (μm) in length ranges between 15.69 – 22.95, while the width ranges between 8.47 – 12.95.

**Table 1.** Colonial morphological characters of eight *Botryodiplodia theobromae* isolates.

Location	Isolates	Colony growth rate after 72 hours	Colony texture and colour on PDA after 72 hours	Brownish pigmentation in PDA slant
NIFOR Main Station	Bt.NGD Bt.NOD Bt.NYD Bt.NRD	+++ +++ +++ +++	Large, flat, uniform, dull white changing to grey Large, flat, uniform, white Large, flat, uniform, dull white changing to grey Large, raised, irregular, greyish-white	- + + -
Coconut Garden, Isihor	Bt.IGD Bt.IOD Bt.IYD Bt.IRD	+++ +++ +++ +++	Large, raised, irregular, dull white changing to grey Large, flat, uniform, greyish white Large, flat, uniform, dull white changing to grey Large, raised, irregular, dull white changing to grey	- - - +

Key: Bt ( *Botryodiplodia theobromae)*, Growth rate (+++: very high), Red pigmentation (+: present; -: absent), N: NIFOR, I: Isihor, GD (green dwarf), OD (orange dwarf), YD (yellow dwarf), RD (red dwarf), PDA (potato dextrose agar)

**Table 2.** Groupings of eight *Botryodiplodia theobromae* isolates based on colony texture and colour.

Isolates	Colony texture and colour on PDA after 72 hours
Bt.NGD Bt.NYD Bt.IYD	Large, flat, uniform, dull white changing to grey
Bt.NOD	Large, flat, uniform, white
Bt.NRD	Large, raised, irregular, greyish white
Bt.IOD	Large, flat, uniform, greyish white
Bt.IGD Bt.IRD	Large, raised, irregular, dull white changing to grey

Key: Bt ( *Botryodiplodia theobromae)*; GD (green dwarf), OD (orange dwarf), YD (yellow dwarf), RD (red dwarf).

**Table 3.** *Botryodiplodia theobromae* conidiospore characteristics.

Location	Isolates	Conidiospore characteristics					
		Day of production	Size in Diameter (μm)		Arrangement	Colour	Septation
			Length	Width			
NIFOR Main Station	Bt.NGD Bt.NOD Bt.NYD Bt.NRD	- 63 70 -	- 19.83 15.69 -	- 11.45 8.47 -	- Scattered Clustered -	- Light brown Light brown -	- Septate/aseptate Septate -
Coconut Garden, Isihor	Bt.IGD Bt.IOD Bt.IYD Bt.IRD	70 70 63 -	18.14 22.13 22.95 -	10.89 12.33 12.95 -	Clustered Scattered Scattered -	Light brown Light brown Light brown -	Septate Septate/aseptate Septate/aseptate -

### Molecular identification, bioinformatics and phylogenetic analysis of *Botryodiplodia theobromae*

**Figure 4.**
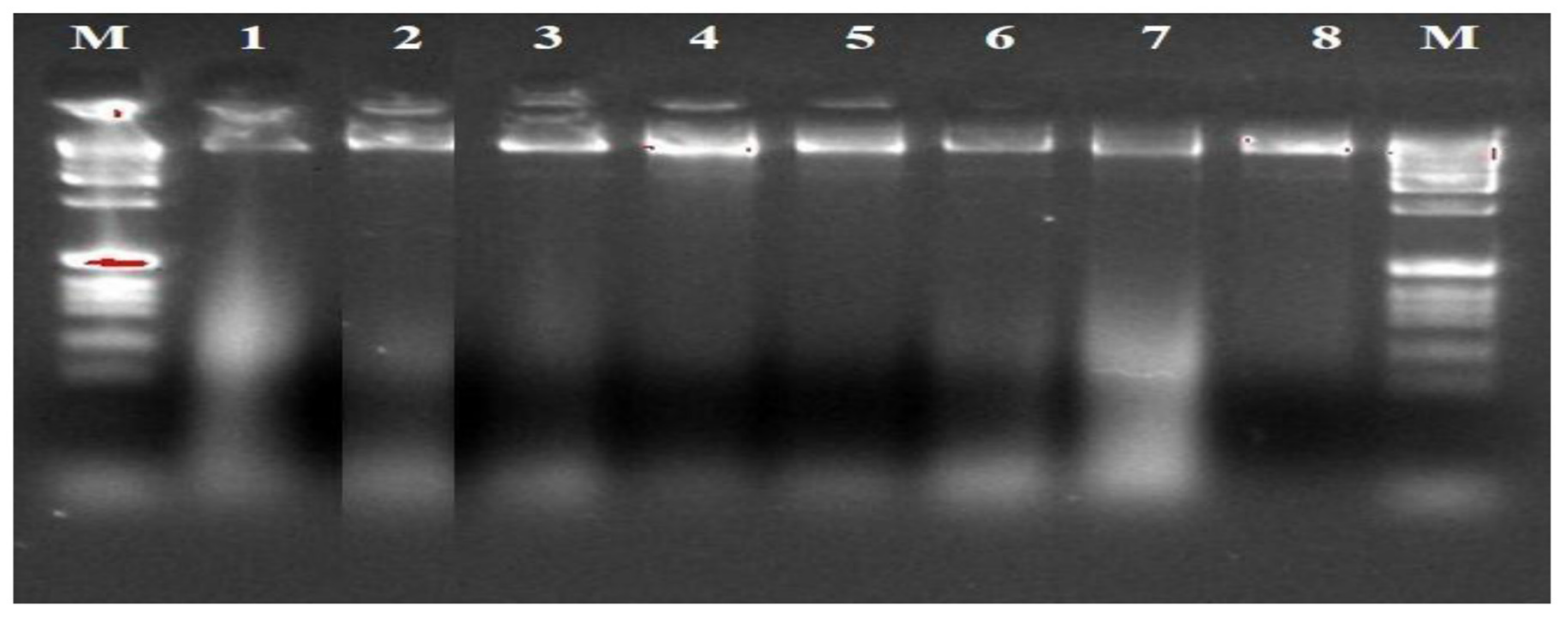
Gel image of genomic DNA of eight isolates.

**Figure 5.**
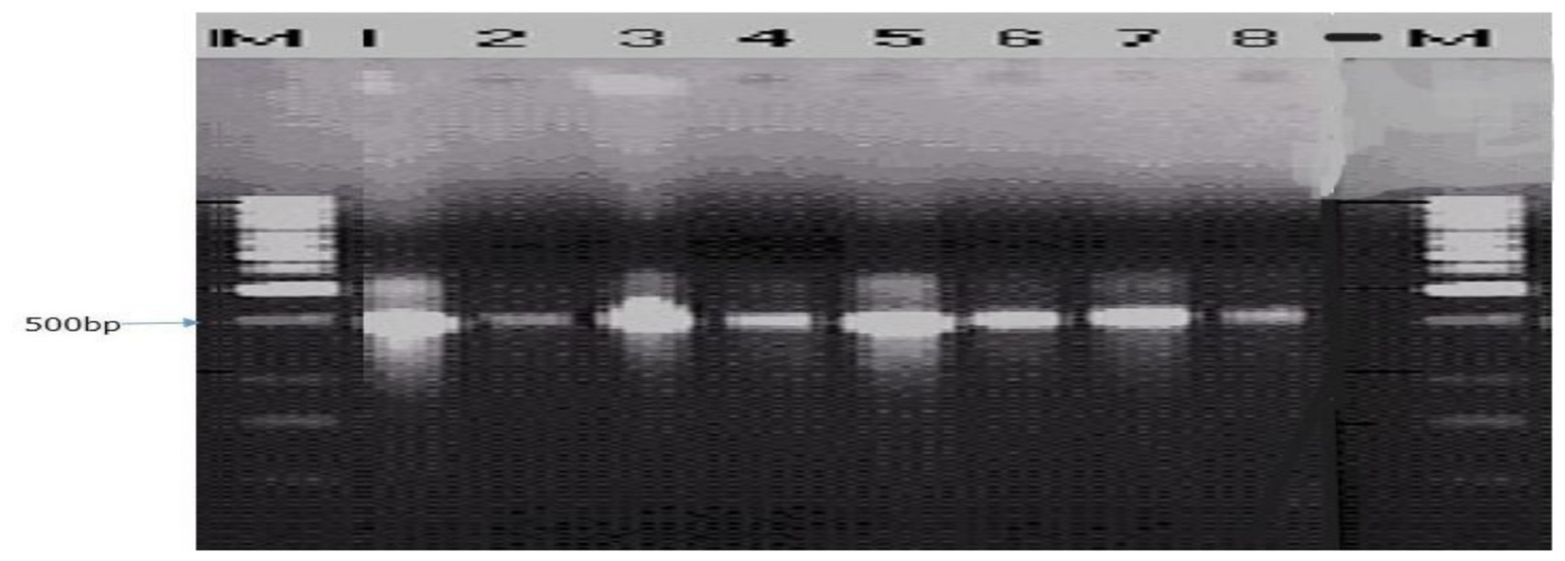
Picture of DNA amplicons of eight isolates using ITS1F/4R primers.

**Figure 6.**
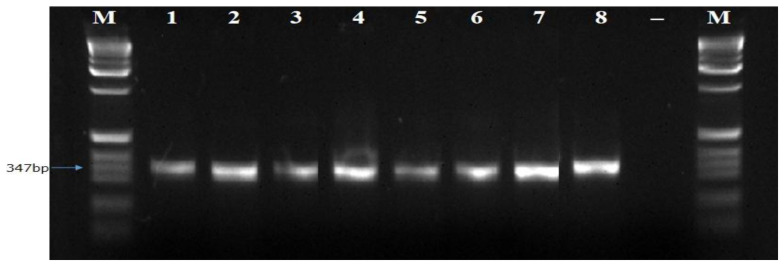
Picture of DNA amplicons of test isolates using Lt347-F/R primers.

**Figure 7.**
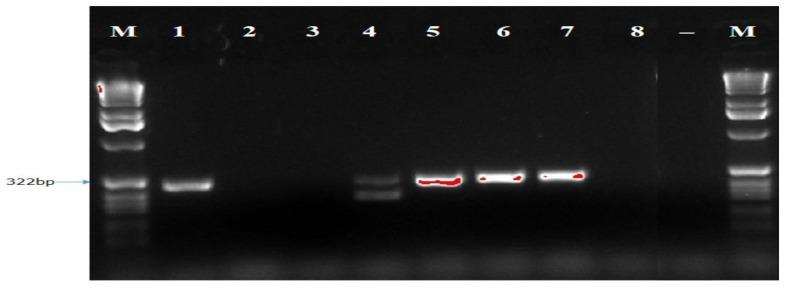
Picture of DNA amplicons of test isolates using Bt2a-F/bR primers.

**Figure 8.**
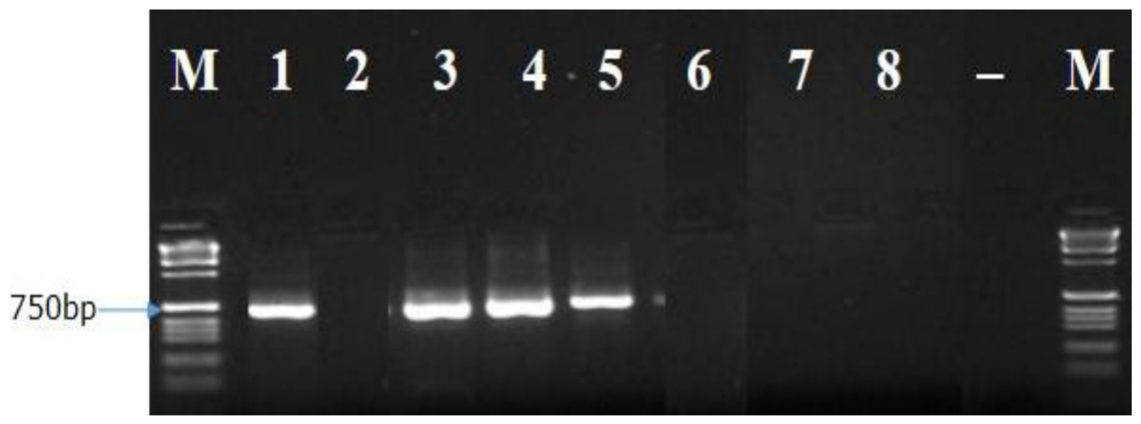
Picture of DNA amplicons of test isolates using EF1-728F/EF2-728R primers.

**Figure 9.1.**
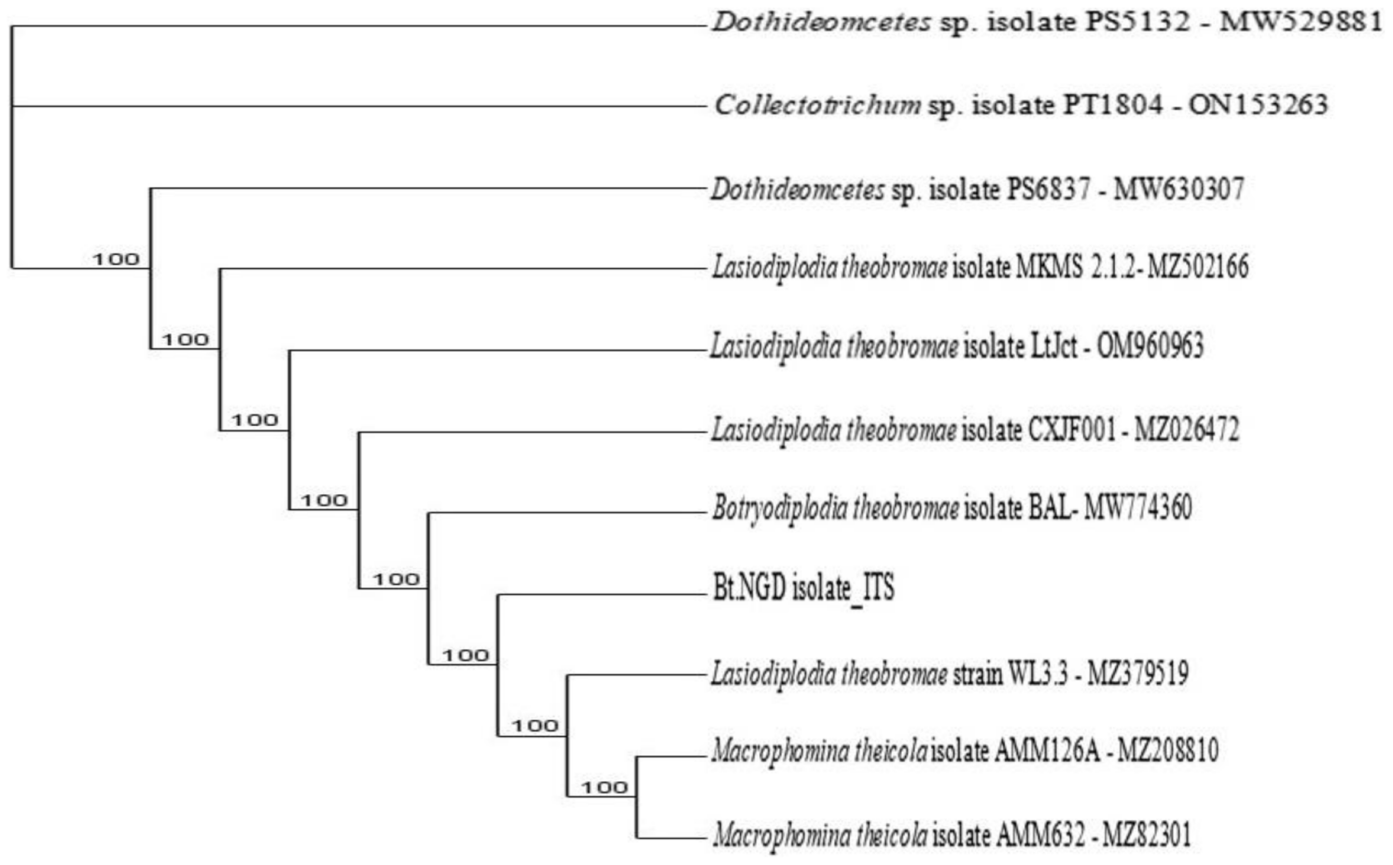
Dendrogram of Bt.NGD using nucleotide sequence of the ITS1F/4R region in NCBI data base.

The ITS1F/4R (universal primer) and Lt347-F/R (specific primer) successfully amplified the DNA of all eight isolates of *B. theobromae* in a PCR prior to diluting the DNA ( Figure 4 and Figure 5). While Bt2aF/bR and EF1-728F/EF2-728R specific primers did not amplify the DNA of all eight isolates of *B. theobromae* in a PCR prior to diluting the DNA. The Bt2aF/bR primer amplified the DNA of isolates Bt.IGD, Bt.NGD, Bt.NYD, and Bt.NRD but was unable to amplify the DNA of other isolates in a PCR ( Figure 6). While EF1-728F/EF2-728R amplified the DNA of isolates Bt. IGD, Bt.IYD, Bt. IRD, and Bt. NGD, it was unable to amplify the DNA of other isolates in a PCR ( Figure 7). Furthermore, the sequence information of the amplified ITS1F/4R region (together with the Lt347-F/R primer) confirms the identity of each of the isolates as *B. theobromae*. The molecular analysis of the ITS1F/4R region also grouped the eight isolates of *B. theobromae* into five groups. Group 1 consists of three isolates: Bt.NYD, Bt.NGD, and Bt.IYD, with accession number MW774360.1; group 2 consists of two isolates: Bt.IGD and Bt.IRD, with accession number OM617921.1; group 3 to 5 consists of one isolate each (Bt.NOD, Bt.NRD, and Bt.IOD), with accession numbers MW282879.1, MZ502166.1, and MW478631.1 ( Table 2 and Table 6). The identified isolates show 100% similarity with respect to each established accession of *B. theobromae* in the NCBI database or gene bank. Additionally, the percentage proportion of individual isolates with *B. theobromae* strains in the NCBI database shows that Group 1 isolates (representing 37.5% of the entire *B. theobromae* population) were more prevalent in both locations, followed by Group 2 isolates (25%), while Groups 3 to 5 represent 12.5%, respectively ( Table 7). Isolates Bt.IOD has high level of genetic distance at site 2 (0.51896) and site 6 (0.61723) in the nucleotide sequence, isolates Bt. IYD has high level of genetic distance at site 6 (0.57171) in the nucleotide sequence, while isolate Bt.IRD also has high level of genetic distance at site 6 (0.56072) in the nucleotide sequence ( Table 7). Other isolates (Bt.NOD, Bt.NRD, Bt.NYD, Bt.IGD, and Bt.NGD) have low genetic distance in the nucleotide sequence. The evolutionary history of each isolate was inferred using the ClustalW algorithm, as disclosed by Kumar *et al*. (2018). The optimal dendogram was constructed for each isolate as illustrated by Kumar *et al*. (2018), Saitou and Nei (1987), and Tamura *et al*. (2004) ( Figure 8, Figure 9.1 to Figure 9.9).

**Table 4.** Coconut fruit population and diseases’ incidence in both locations and growing seasons.

Locations	Growing seasons	No of coconut fruits sampled	No of diseased coconut fruits			PDO
				FR	PNF	
NIFOR Main Station	2021 – 2022	1922	258	13.4	110	148
	2022 – 2023	2536	419	195	224	16.5
	**Grand total**	**4458**	**677**	**305**	**372**	**15.2**
Coconut Garden, Isihor	2021 – 2022	2238	266	128	138	11.9
	2022 – 2023	2068	255	148	107	12.3
	**Grand total**	**4306**	**521**	**276**	**245**	**12.1**

Key: FR (fruit rot), PNF (Premature nut fall), PDI (percentage of disease incidence).

**Table 5.** Analysis of genomic DNA extracted from isolates of *Botryodiplodia theobromae* using nano drop spectrophotometer.

SAMPLES	DNA CONC (ng/µl)	A _260_	A _280_	A _260/280_
Isolate 1	1273.3	26.446	14.395	1.837
Isolate 2	1827.0	35.539	18.291	1.943
Isolate 3	3068.0	61.360	31.041	1.980
Isolate 4	1177.3	43.546	21.387	2.036
Isolate 5	1032.5	40.651	21.985	1.849
Isolate 6	1580.2	31.604	15.661	2.020
Isolate 7	803.4	40.068	20.969	1.911
Isolate 8	1533.7	30.675	15.285	2.007

**Table 6.** Grouping of eight *Botryodiplodia theobromae* isolates based on molecular identity.

Groups	Isolates	Relationship to isolates in NCBI gene bank	Accession number	% proportion of individual strains
I	Bt.NYD Bt.NGD Bt.IYD	*Botryodiplodia theobromae* isolate BAL2	MW774360	37.5
II	Bt.IGD Bt.IRD	*Botryodiplodia theobromae* isolate LSB-1	OM617921	25
III	Bt.NOD	*Botryodiplodia theobromae* isolate MRR-030	MW282879	12.5
IV	Bt.NRD	*Botryodiplodia theobromae* isolate MKMS 2.1.2	MZ502166	12.5
V	Bt.IOD	*Botryodiplodia theobromae* isolate C18	MW478631	12.5

**Table 7.** Average genetic distance of eight *Botryodiplodia theobromae* from two locations.

**Isolates**	**Site 1**	**Site 2**	**Site 3**	**Site 4**	**Site 5**	**Site 6**	**Site 7**
Bt.NGD	0.00000						
Bt.NOD	0.00795						
Bt.NYD	0.00000	0.00996					
Bt.NRD	0.00198	0.00998	0.00198				
Bt.IGD	0.01392	0.01002	0.01793	0.00800			
Bt.IOD	0.57853	0.51896	0.57853	0.55689	0.61723		
Bt.IYD	0.00198	0.01992	0.00198	0.00000	0.00798	0.57171	
Bt.IRD	0.01408	0.00203	0.01408	0.01411	0.02434	0.56072	0.02218

Key: The genetic distance ranges from 0.00198 to 0.61723. The compositional distance correlate with the number of differences between sequences. High genetic distance: 0.5 above; Low genetic distance: 0.5 below.

**Figure 9.2.**
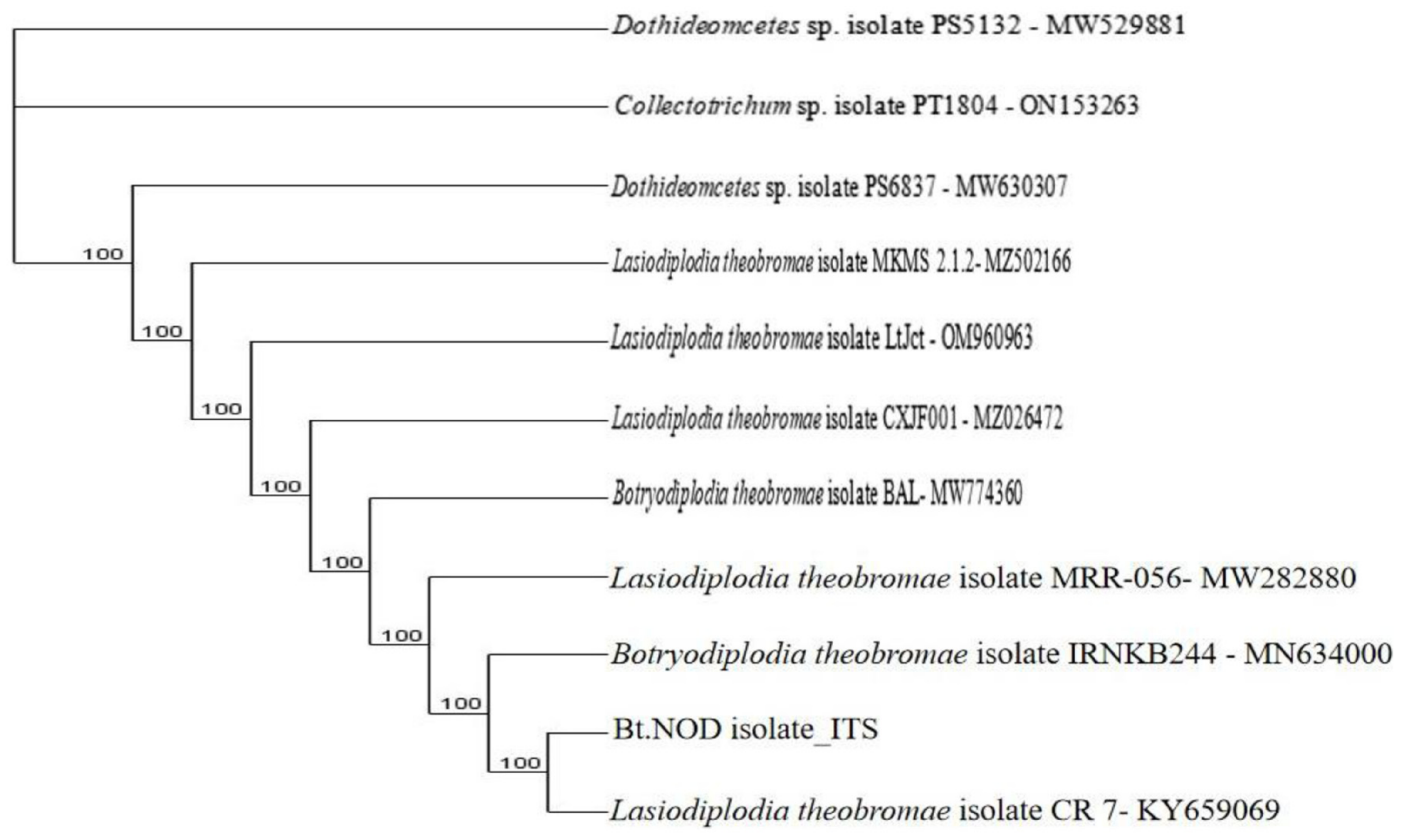
Dendrogram of Bt.NOD using nucleotide sequence of the ITS1F/4R region NCBI data base.

**Figure 9.3.**
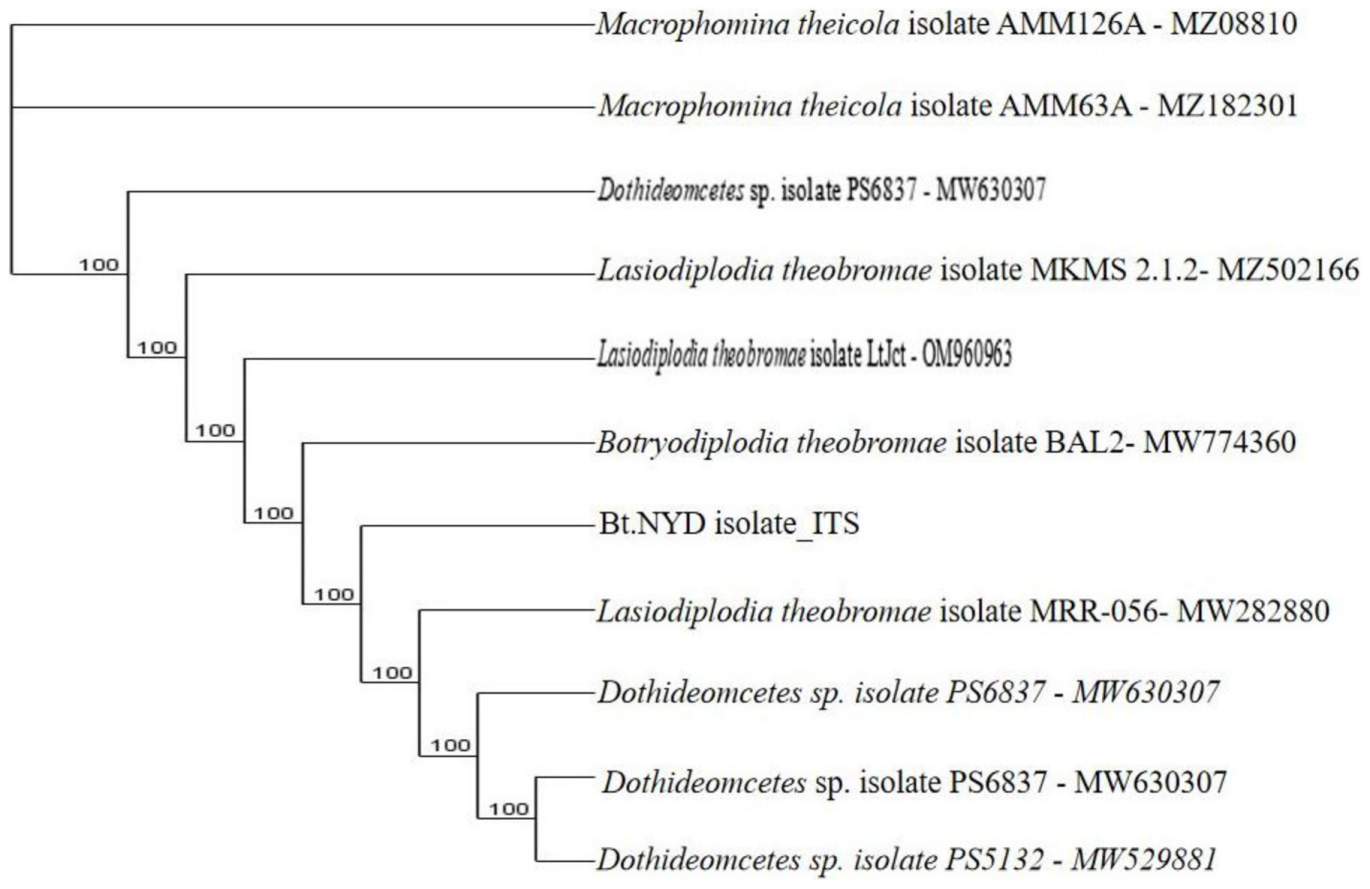
Dendrogram of Bt.NYD using nucleotide sequence of the ITS1F/4R region in NCBI data base.

**Figure 9.4.**
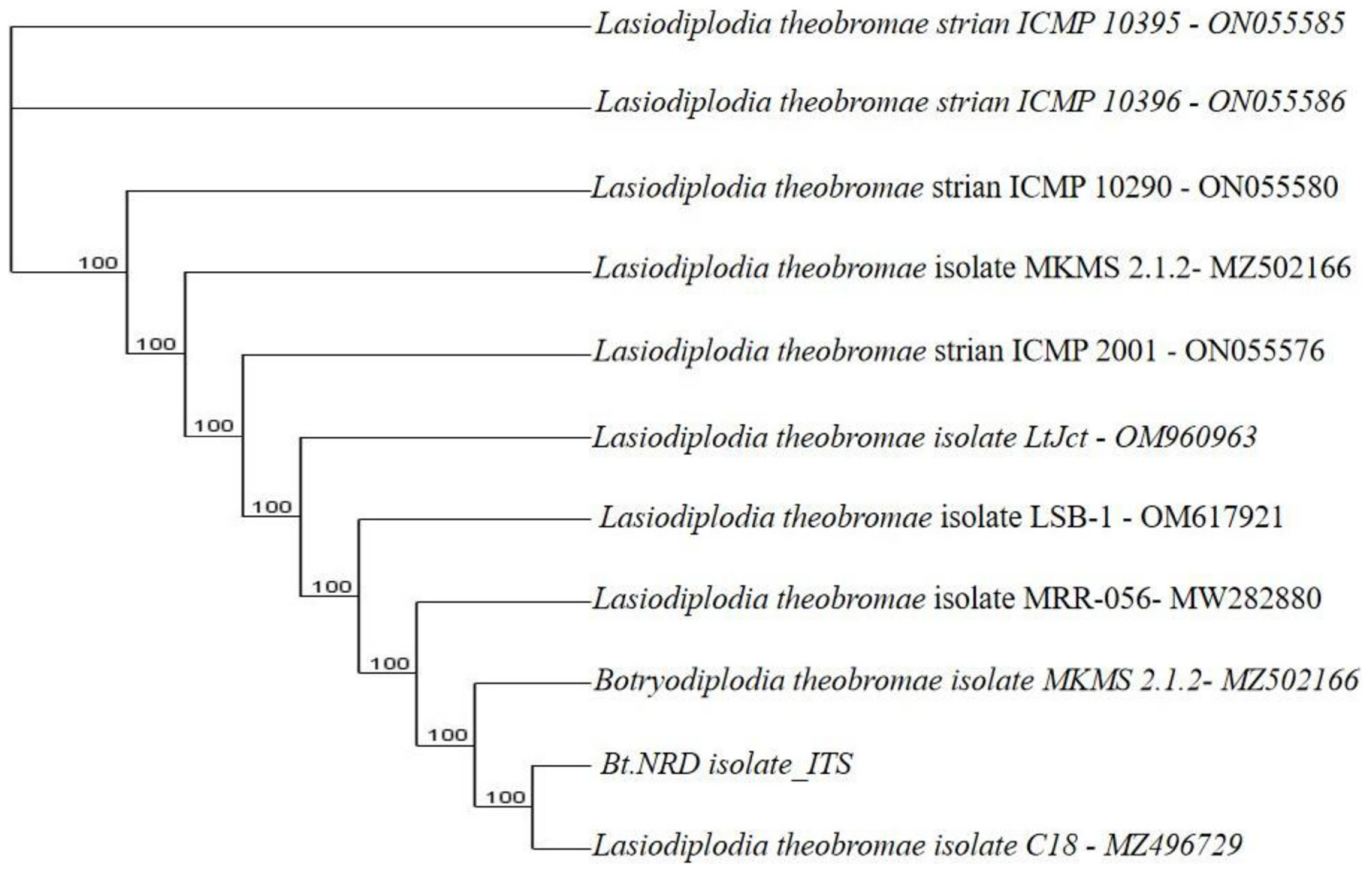
Dendrogram of Bt.NRD using nucleotide sequence of the ITS1F/4R region in NCBI data base.

**Figure 9.5.**
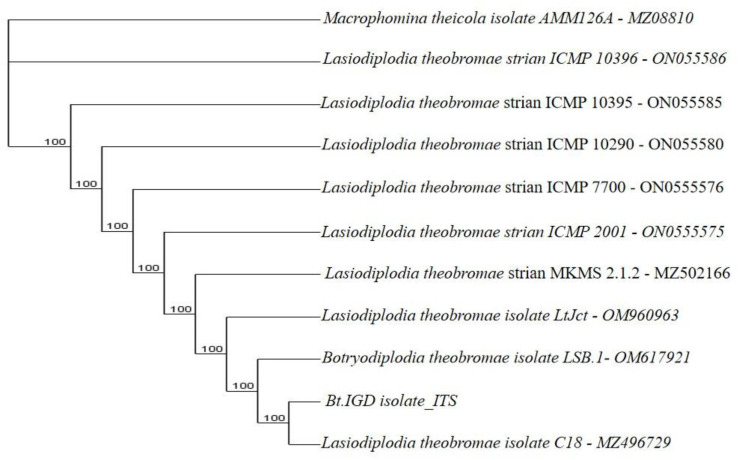
Dendrogram of Bt.IGD using nucleotide sequence of the ITS1F/4R region in NCBI data base.

**Figure 9.6.**
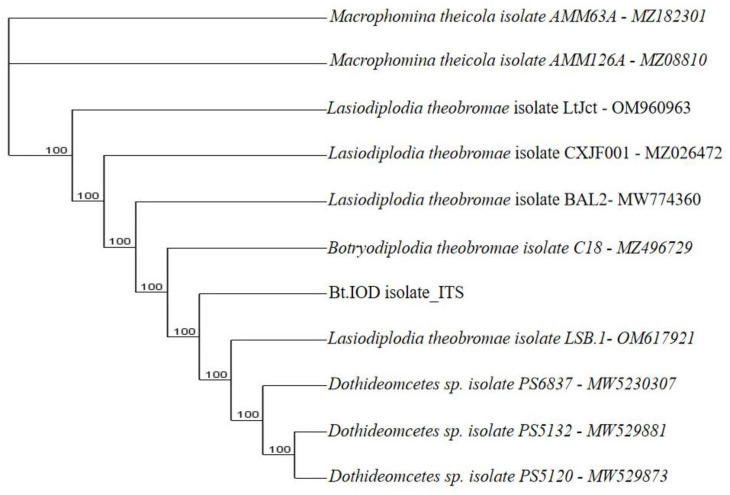
Dendrogram of Bt.IOD using nucleotide sequence of the ITS1F/4R region in NCBI data base.

**Figure 9.7.**
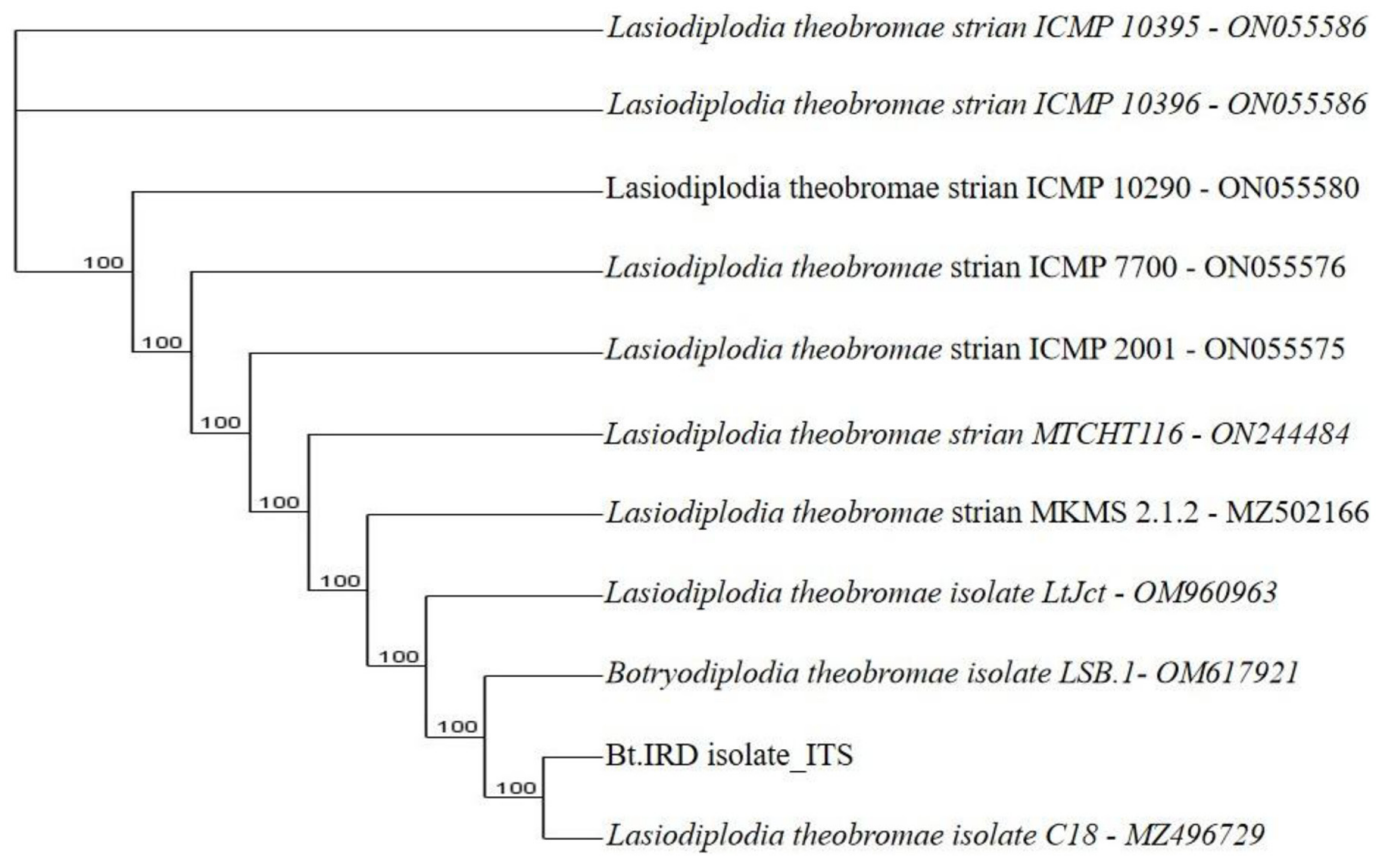
Dendrogram of Bt.IYD using nucleotide sequence of the ITS1F/4R region in NCBI data base.

**Figure 9.8.**
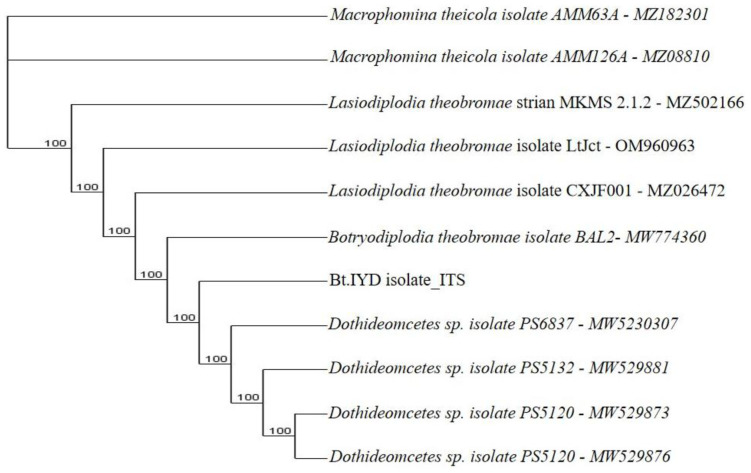
Dendrogram of Bt.IRD using nucleotide sequence of the ITS1F/4R region in NCBI data base.

**Figure 9.9.**
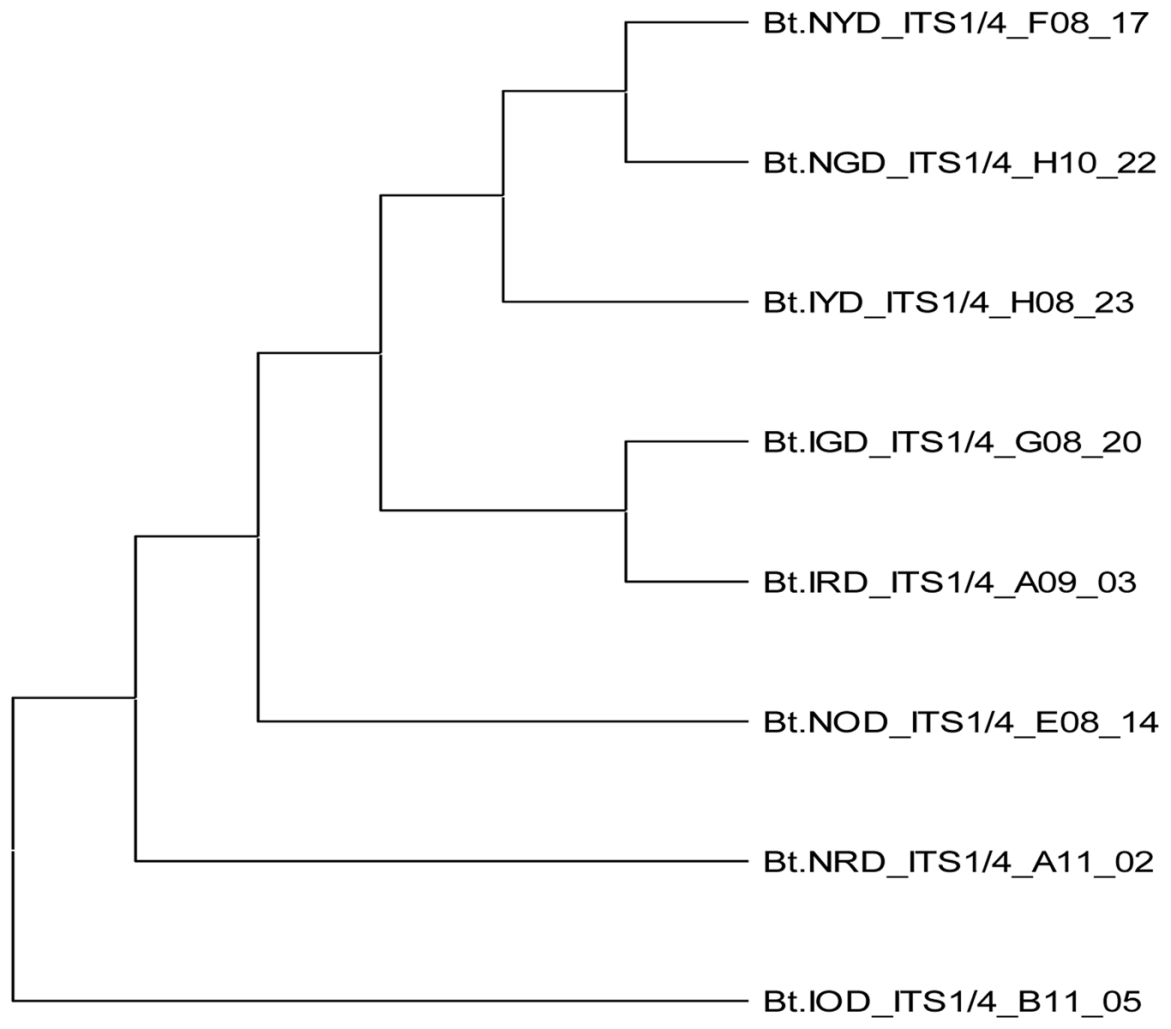
Dendrogram obtained from the combined data set of eight *Botryodiplodia theobromae* isolates.

## Discussion

As previously mentioned, this study was conducted in two major coconut-growing locations in Edo State, Nigeria. The fungal pathogen was isolated from diseased coconut fruits showing signs of rot and fallen nuts ( Phipps & Porter, 1998). The colony description of each fungal pathogen together with the microscopy observation were used for the presumptive identification of the eight isolates of *B. theobromae* ( Ashokkumar *et al*., 2018). In addition, after 72 hours, the eight isolates of *B. theobromae* exhibit different colony pigments and textures according to the colonial morphological features that were determined in the study ( Adeniyi *et al*., 2016). The colony pigments of *B. theobromae* isolates varied from white (Bt. NOD), to dull white changing to grey (Bt.NRD, Bt.NGD, Bt.NYD, and Bt.IYD), to greyish white (Bt.IOD, Bt.IRD, and Bt.IGD). The mycelia topography were either raised or flat, while the mycelia edge were either irregular or uniform. The colony pigments and textures grouped all eight isolates of *B. theobromae* into five categories ( Table 2). Conidiospore arrangement were either clustered (Bt.NYD and Bt.IGD) or scattered (Bt.NOD, Bt.IOD and Bt.IYD). Conidiospore size in length ranges between 15.69 to 22.95 μm, while the width ranges between 8.47 to 12.95 μm. The conidiospore are light brown, initially hyaline, mostly septate, with few aseptate ( Figure 3). Based o the numerical figures obtained from the study, isolates from Coconut Garden, Isihor, had conidiospore which numbers were greater that than that obtained from NIFOR Main Station. Studies on the colonial morphological features align with the work of Adeniyi *et al*. (2016). The colony growth rate of the eight isolates was relatively rapid after 72 hours of the incubation process, suggesting that they had grown quickly ( Table 1). After being incubated in PDA for 72 hours, all eight isolates covered the area of the Petri plate. It is postulated that *B. theobromae* fastidious nature makes them to colonize susceptible host tissues easily ( Ashokkumar *et al*., 2018; Dheepa *et al*., 2018).

To confirm the identity of the eight *B. theobromae* isolates using molecular technique, one universal primer (ITS1F/4R) and three specific primers (Lt347-F/R, Bt2aF/bR, EF1-728F/EF2-728R) were employed to amplify the genes of *B. theobromae* isolates. The ITS1F/4R and Lt347-F/R primers successfully amplified the DNA of all eight *B. theobromae* isolates in a PCR. But the two other specific primers (Bt2aF/bR, EF1-728F/EF2-728R) were unable to amplify the DNA of all eight *B. theobromae* isolates in a PCR. Additionally, the amplified ITS1/4 region of the eight isolates was sequenced and blasted in the NCBI database, which shows 100% similarity with *B. theobromae* (accession numbers: MW774360.1, OM617921.1, MW282879.1, MZ502166.1, and MG778942.1) in the NCBI database. Also, the ability of the specific primer (Lt347-F/R) to amplify the DNA of *B. theobromae* confirms the identity of all eight isolates examined in the study. Hence, in this study, the eight fungal isolates responsible for the fruit rot and premature nut fall diseases of coconut were identified and confirmed as *B. theobromae*. The ITS1F/4R primer was successfully utilized to amplify the ITS1/4 gene and for the quick identification of various isolates of *B. theobromae*, which is in line with different investigations ( Ashokkumar *et al*., 2018; Bharanideepan, 2013; Munirah *et al*., 2017). However, Xu *et al*. (2016) demonstrated that *B. theobromae* could also be identified using a species-specific (Lt347-F/R) primer. In addition, based on ITS1/4 sequence information, Bt.NGD, Bt.NYD, and Bt.IYD isolates are genetically similar, as all three isolates have similar ITS1/4 sequence information with *B. theobromae* isolate BAL2 with accession number MW774360.1. Two isolats (Bt.NGD, Bt.NYD) from the NIFOR location and one isolate (Bt.IYD) from Isihor were grouped into Group 1. This simply suggests these isolates do coexist and have a connection in both places. Because of this, they are more common in both places. While Bt.IGD and Bt.IRD isolates are genetically similar, the two isolates have similar ITS1/4 sequence information to *B. theobromae* isolate LSB-1 with accession number OM617921.1. Both isolates were in the same location (Isihor). This helps explain why they are so common in the Isihor area. Bt.NOD, Bt.NRD, and Bt.IOD isolates have no ITS1/4 similarity with each other. Also, they have no similarity (in terms of the ITS1/4 gene) to the other isolates mentioned previously. In addition, Bt.NOD isolate has similar ITS1/4 sequence information with *B. theobromae* isolate MRR-030 with accession number MW282879.1, Bt.NRD isolate has similar ITS1/4 sequence information with *B. theobromae* isolate MKMS 2.1.2 with accession number MZ502166.1, and Bt.IOD isolate has similar ITS1/4 sequence information with *B. theobromae* isolate C18 with accession number MG778942.1 ( Table 7). The ITS1/4 sequence information and phylogenetic analysis grouped all eight isolates of *B. theobromae* into five categories ( Table 7 and Figure 1). Coincidentally, the cultural and molecular techniques were able to group the eight isolates of *B. theobromae* into five groups. Group 1 consists of 3 isolates (Bt.NYD, Bt.NGD, and Bt.IYD with accession number MW774360.1); Group 2 consists of 2 isolates (Bt.IGD and Bt.IRD with accession number OM617921.1); and Groups 3 to 5 consist of 1 isolate each (Bt.NOD, Bt.NRD, and Bt.IOD with accession numbers MW282879.1, MZ502166.1, and MG778942.1, respectively). Group 1 isolates (37.5%) were more prevalent in both locations, followed by group 2 isolates (25%), while groups 3 to 5 represent 12.5%, respectively. The prevalence of Group 1 isolates in both locations indicates that these isolates are mostly responsible for the coconut fruit rot and premature nut fall diseases, followed by Group 2 isolates and Groups 3 – 5 isolates. Isolates Bt.IOD, Bt.IYD, and Bt.IRD have a high level of genetic distance at specific sites in their nucleotide sequence, while isolates Bt.NOD, Bt.NRD, Bt.NGD, Bt.IGD, and Bt.NYD have a low genetic distance in their nucleotide sequence. The substantial amount of inherited distance through genes is proportional to the extent of disparities in the nucleotide sequences in each of the three isolates, even when the substitution patterns of the nucleotide sequences are homogeneous among the lineages of each isolate.

## Conclusion

*Botryodiplodia theobromae* causing the fruit rot and premature nut fall diseases of coconut was identified based on its cultural, morphological and molecular characteristics. Although *B. theobromae* isolates showed similarity and variation in their cultural, morphological and molecular characters. The colony colour, ITS1/4 gene sequence information and phylogenetic analysis grouped all eight isolates of *B. theobromae* into five categories, as these characters assisted in the identification and characterization of *B. theobromae*. The study has provided a guide for proper identification of *B. theobromae* required for the proper control of this pathogen in coconut producing areas. More so, coconut growers should intensify regular fungal inspections and contact expert phytopathologist for proper guidance on how to adequately control the disease’s condition in coconut producing areas.

## Transparency

The authors state that the manuscript is honest, truthful, and transparent, that no key aspects of the investigation have been omitted, and that any differences from the study as planned have been clarified. This study followed all writing ethics.

## Ethics and consent

Ethical approval and consent were not required.

## Data Availability

**Mendeley:** Cultural-morphology and molecular analysis of *Botryodiplodia theobromae*, a pathogen of coconut fruit. Doi: 10.17632/53ns2fcgps.1 ( https://data.mendeley.com/datasets/53ns2fcgps/1) ( Ekhorutomwen, 2025) This project contains the following underlying data: •  Osayomore *et al*_Manuscript.docx •  Osayomore_Unedited_Data.docx Data are available under the terms licensed under a Creative Commons Attribution 4.0 International licence.

## Accession numbers

NCBI Gene: *B. theobromae* isolated from diseased green and yellow dwarf coconut fruit [ *Coco nucifera* (coconut)]. Accession number MW774360.1. https://www.ncbi.nlm.nih.gov/nuccore/MW774360.1/

NCBI Gene: *B. theobromae* isolated from diseased green and red dwarf coconut fruit [ *Coco nucifera* (coconut)]. Accession number OM617921.1. https://www.ncbi.nlm.nih.gov/nuccore/OM617921.1/

NCBI Gene: *B. theobromae* isolated from diseased orange dwarf coconut fruit [ *Coco nucifera* (coconut)]. Accession number MW282879.1. https://www.ncbi.nlm.nih.gov/nuccore/MW282879.1

NCBI Gene: *B. theobromae* isolated from diseased red dwarf coconut fruit [ *Coco nucifera* (coconut)]. Accession number MZ502166.1. https://www.ncbi.nlm.nih.gov/nuccore/MZ502166.1

NCBI Gene: *B. theobromae* isolated from diseased orange dwarf coconut fruit [ *Coco nucifera* (coconut)]. Accession number MW478631.1. https://www.ncbi.nlm.nih.gov/nuccore/MW478631.1
