# Supplementary material for: Cultural-morphology and molecular analysis of Botryodiplodia theobromae, a pathogen of coconut fruit
Source: Open Res Eur. 2025 Nov 12;5:140. Originally published 2025 May 21. [Version 2] doi: 10.12688/openreseurope.19692.2 (PMC12754368; doi:10.12688/openreseurope.19692.2)
Supplement: Peer review history

# Reviewer report: Reviewer response for version 2

**DOI:** 10.21956/openreseurope.23306.r64025

**Published:** 2025-12-30

**Authors:** Lellapalli Rithesh

**Affiliations:** 1 PLANT PATHOLOGY, JC DIWAKR REDDY AGRICULTURAL COLLEGE (ANGRAU) (Ringgold ID: 363676), TADIPATRI, ANDHRA PRADESH, India

Dear Editor, I am satisfied with the new version of the manuscript

 Thank you

Is the study design appropriate and does the work have academic merit?

Yes

Is the work clearly and accurately presented and does it cite the current literature?

Partly

If applicable, is the statistical analysis and its interpretation appropriate?

Not applicable

Are all the source data underlying the results available to ensure full reproducibility?

Yes

Are the conclusions drawn adequately supported by the results?

Partly

Are sufficient details of methods and analysis provided to allow replication by others?

Partly

Reviewer Expertise:

Plant Pathology, Mycology, Coconut, Host Pathogen Interactions

I confirm that I have read this submission and believe that I have an appropriate level of expertise to confirm that it is of an acceptable scientific standard.

# Reviewer report: Reviewer response for version 2

**DOI:** 10.21956/openreseurope.23306.r64024

**Published:** 2025-11-28

**Authors:** Egya N. Yankey

**Affiliations:** 1 Coconut Research Program, Council for Scientifc and Industrial Research (CSIR), Sekondi, Ghana

I am satisfied with the responses from the authors; I therefore recommend that the manuscript be accepted for indexing.

Is the study design appropriate and does the work have academic merit?

Yes

Is the work clearly and accurately presented and does it cite the current literature?

Yes

If applicable, is the statistical analysis and its interpretation appropriate?

Not applicable

Are all the source data underlying the results available to ensure full reproducibility?

Yes

Are the conclusions drawn adequately supported by the results?

Partly

Are sufficient details of methods and analysis provided to allow replication by others?

Partly

Reviewer Expertise:

Molecular Plant Pathoolgist, Phytoplasma diseases of coconuts.

I confirm that I have read this submission and believe that I have an appropriate level of expertise to confirm that it is of an acceptable scientific standard.

# Reviewer report: Reviewer response for version 1

**DOI:** 10.21956/openreseurope.21300.r60137

**Published:** 2025-10-01

**Authors:** Lellapalli Rithesh

**Affiliations:** 1 PLANT PATHOLOGY, JC DIWAKR REDDY AGRICULTURAL COLLEGE (ANGRAU) (Ringgold ID: 363676), TADIPATRI, ANDHRA PRADESH, India

This manuscript reports on the cultural, morphological, and molecular characterization of *Botryodiplodia theobromae* isolates obtained from diseased coconut fruits showing fruit rot and premature nut fall in two locations in Edo State, Nigeria. Eight isolates were collected from four coconut varieties. The authors examined colony growth, pigment production, conidiospore characteristics, and used one universal primer (ITS1F/4R) and three species-specific primers (Lt347-F/R, Bt2aF/bR, and EF1-728F/EF2-728R) for PCR amplification. ITS sequences were analyzed and compared to NCBI entries, grouping the isolates into five genetic clusters. The study concludes that the pathogen causing fruit rot and premature nut fall in these locations is *B. theobromae*, and that molecular and cultural characterization helps in early diagnosis and management.

 Clarity and literature:

 The paper is generally clear and cites many current and classical references on *B. theobromae*. However, the introduction repeats some points (host range, endophytic nature) and could be condensed. The plain language summary is very brief and could better explain why the study matters to farmers and plant health authorities. References are up to date but a few key regional or methodological papers could still be added, for example on the link between *Lasiodiplodia* genetic groups and virulence.

 Study design and merit:

 The design combining morphology with molecular analysis is appropriate and has academic merit. The use of multiple primers strengthens the conclusions. Including isolates from two locations and different coconut varieties adds value.

 Methods and replicability:

 The methods are described in detail, which is positive, but sometimes procedural minutiae (exact volumes of disinfectants) obscure key information. It should be stated clearly which primers succeeded or failed and why only ITS data were used for phylogenetic analysis. Lane labels in the gel images should be clearer. Explaining how “site 1…site 7” in the genetic distance table was defined will help others replicate the analysis. With these clarifications, replication would be straightforward.

 Statistical analysis:

 No formal statistical tests were performed; the study is descriptive. This is acceptable for a diagnostic/characterization paper but could be strengthened by simple quantitative comparisons (e.g. conidiospore size or growth rate differences between groups).

 Specific points to address to make the article scientifically sound

1. Condense and clarify the introduction to avoid repetition and explicitly state the research questions or hypotheses.
2. Clarify the molecular methods ****why only ITS data were used for tree building, and what “prior to diluting DNA” means.
3. Improve figure and table presentation ****combine gel images into one figure with lane labels; explain “site” numbers in the genetic distance table; add a scale bar and, if possible, bootstrap values to the dendrogram.
4. Strengthen the discussion by interpreting patterns (prevalence of groups, pigment production, genetic distance) rather than only restating results. Explain how findings might influence management strategies.
5. Polish language and spelling ****shorten long sentences, ensure consistent terminology (septate vs. sepatate, conidiospores vs. conidiospore).
6. Expand the plain language summary to highlight why early diagnosis matters and how farmers could benefit.

Is the study design appropriate and does the work have academic merit?

Yes

Is the work clearly and accurately presented and does it cite the current literature?

Partly

If applicable, is the statistical analysis and its interpretation appropriate?

Not applicable

Are all the source data underlying the results available to ensure full reproducibility?

Yes

Are the conclusions drawn adequately supported by the results?

Partly

Are sufficient details of methods and analysis provided to allow replication by others?

Partly

Reviewer Expertise:

Plant Pathology, Mycology, Coconut, Host Pathogen Interactions

I confirm that I have read this submission and believe that I have an appropriate level of expertise to confirm that it is of an acceptable scientific standard, however I have significant reservations, as outlined above.

# Response

**Published:** 2025-10-02

**Authors:** OSAYOMORE EKHORUTOMWEN

**Affiliations:** Crop Production and Protection, Not Applicable, Benin City, Edo, Nigeria

I have provided the necessary information in the updated manuscript to the best of my ability.

# Reviewer report: Reviewer response for version 1

**DOI:** 10.21956/openreseurope.21300.r54733

**Published:** 2025-06-24

**Authors:** Egya N. Yankey

**Affiliations:** 1 Coconut Research Program, Council for Scientifc and Industrial Research (CSIR), Sekondi, Ghana

The manuscript is well-written and examines an important topic that is relevant to coconut production. **While I recommend it for indexing, there are a few drawbacks to the study that must be addressed.**

 1.The authors mention the fungus *Botryodiplodia theobromae* as the cause of fruit rot and nut drop. Fruit rot and nut drop are associated with several biotic and abiotic factors, including lethal yellowing diseases, Phytophthora bud rot, high temperatures, and dry spells, among others. The authors should therefore correct the statement throughout the manuscript to read that the fungus is ‘associated’ with the condition rather than being the cause.

 2. No controls were included in the study. Samples from uninfected coconut fruits from the sampling sites should have been included in both the cultures and in the PCR analysis. The authors should clarify why this was not done.

 3. In the gel pictures, there is a blank well after the eighth lane: is this an empty well or a negative control? If it is a control, kindly indicate it and state what the control test was (water?).

 4. In the result section, under the molecular identification, the authors mention ‘prior to diluting the DNA’. Please, what is the relevance of this statement?

 5. The statement ‘DNA profile’ under Data analysis should read ‘DNA sequences’

 6. A phylogenetic tree including sequences of the fungus from other plant species will be informative. An outgroup for rooting the tree should also be included.

 7. A pictorial of the symptoms, fruit rot, and nut fall will be informative and should be included in the article.

 8. The authors should add a couple of disease management strategies for the pathogen in the conclusion section.

Is the study design appropriate and does the work have academic merit?

Yes

Is the work clearly and accurately presented and does it cite the current literature?

Yes

If applicable, is the statistical analysis and its interpretation appropriate?

Not applicable

Are all the source data underlying the results available to ensure full reproducibility?

Yes

Are the conclusions drawn adequately supported by the results?

Partly

Are sufficient details of methods and analysis provided to allow replication by others?

Partly

Reviewer Expertise:

Molecular Plant Pathoolgist, Phytoplasma diseases of coconuts.

I confirm that I have read this submission and believe that I have an appropriate level of expertise to confirm that it is of an acceptable scientific standard, however I have significant reservations, as outlined above.

# Response

**Published:** 2025-10-02

**Authors:** OSAYOMORE EKHORUTOMWEN

**Affiliations:** Crop Production and Protection, Not Applicable, Benin City, Edo, Nigeria

Response to comment 2: We isolated the test pathogen from diseased coconut fruit, but we didn't isolate fungi from healthy coconut fruits. Our study was not focused on the microbial community of coconut fruit.  

 Response to comment 3: There gel pictures only used markers as the control.  

 Response to comment 4. the statement has been corrected.  

 Response to comment 6: Requested information has been provided in the updated manuscript.

 Response to comment 7. A pictorial of the symptoms, fruit rot and nut fall has been provided.

 Response to comment 8. This information has been provided in the updated manuscript.   Thank you so much for your review.
